# A novel dual feedwater circuit for a parabolic trough solar power plant

**DOI:** 10.1038/s41598-023-33829-1

**Published:** 2023-05-08

**Authors:** Wisam Abed Kattea Al-Maliki, Sajda S. Alsaedi, Hayder Q. A. Khafaji, Falah Alobaid, Bernd Epple

**Affiliations:** 1 Institut Energiesysteme und Energietechnik, TU Darmstadt, Otto-Berndt-Straße 2, 64287 Darmstadt, Germany; 2 Mechanical Engineering Department, University of Technology-Iraq, Ministry of Higher Education and Scientific Research, Baghdad, Iraq; 3 Department of Electromechanical Engineering, University of Technology-Iraq, Baghdad, Iraq

**Keywords:** Energy storage, Renewable energy, Solar energy, Solar thermal energy, Energy science and technology, Engineering, Mechanical engineering

## Abstract

The validated dynamic model of a parabolic trough power plant (PTPP) is improved by the combination of a new feedwater circuit (feedwater/HTF circuit) and a reference feedwater circuit (feedwater/steam circuit) as well as the development of the steam turbine model. Such design represents the first effort of research to utilize a dual feedwater circuit inside the PTPP to increase the power output in the daylight from 50 to 68 MW_el_ and raise night operating hours at a lower cost. The purpose of increasing the operating night hours at a power (48 MW_el_) as in the reference PTPP is to get rid of the fossil fuel backup system and rely only on the absorbed solar energy and the stored energy in the molten salt. During daylight hours, the feedwater circuit is operated using Feedwater/HTF. In the transient period, the feedwater/HTF circuit will gradually be closed due to a decrease in solar radiation. Furthermore, the rest of the nominal feedwater mass flow rate (49 kg/s) is gradually replenished from the feedwater/steam circuit. After sunset, the entirety of the feedwater is heated based on the steam extracted from the turbine. The purpose of this improvement is to raise the number of nightly operational hours by reducing the nominal load from 61.93 to 48 MW_el_ as a result of low energy demand during the evening hours. Therefore, a comparison study between the reference model and this optimization (optimization 2) is conducted for clear days (26th–27th/June and 13th–14th/July 2010) in order to understand the influence of dual feedwater circuit. The comparison indicates that the operational hours of the power block (PB) will be obviously increased. Moreover, this improvement reduces based on the fossil fuel system at night. As the last step, an economic analysis was performed on the costs of the referenced and the optimized PTPP as a function of the levelized energy cost (LEC). The results illustrate that the specific energy cost of a PTPP with 7.5 h of storage capacity is lowered by about 14.5% by increasing the output of the PTPP from 50 to 68 MW_el_.

## Introduction

The use of concentrated solar power (CSP) for generating electricity is a key step in the direction of environmentally sustainable growth and offers a highly preferable alternative against atmospheric degradation^1, 2^. CSP technologies for achieving high-temperature are used. CSP facilities are focusing on direct solar irradiance on narrow areas, allowing high-temperature to be achieved. In CSP technologies, a parabolic trough (PT) is able to be regarded as a perfected technology under CSP plants, which has also demonstrated its economic viability^3–17^. For instance, the PTPPs are capable of reaching temperatures approaching 395 °C^18^. Such power plants have a thermal storage system (TSS) for continuous power generation through hours in the absence of sunlight^6^.

To complement more experimental investigations, simulation modelling of PTPP supports the comprehension of the system operation, its potentials, and constraints. Enhancements and reconfigurations of power systems typically start with steady-state modelling of processes. As opposed to this, using dynamic modelling enables users and engineers to develop better operating strategies and process control suggestions^17, 19^. So far, various kinds of experimentation for the modelling and simulation of PTPP have been carried out. Achievement model validity and testing of various operational approaches constituted the principal purposes behind these efforts. In the following, a broad survey of dynamic modelling investigations related to the PTPP is reviewed.

Yuanjing et al. ^1^ suggested improving the 30 MW parabolic aqueduct solar thermal power plant. They specified a model for the entire plant performance. A commercial program Ebsilon to build the simulation models of the (SEGS VI) Plant was computed. Further, a performance analysis of the twain plants in a specific design and operating conditions was conducted. In addition, they assessed all implementation factors of the SEGS VI with an enhanced system. The findings reveal that the efficiency of the solar field boosts around 0.52% and the entire performance of plants boosts around 0.22% at the operating atmospheres. At the same time, the collector numbers of the solar aspect increase, which get a great application chance. Liu et al.^20^ developed a modal predictive regulator to merge the true power load with information for predictive climate data, to reduce cumulative coal consumption on a distinct day and particular duration time. Further, they conducted a simulation for successive ten days to see the advantages and operating procedures of the model predictive regulator. A comparison between the traditional regulator and load predictions was performed, a particular day simulation displays, that the coal-consuming reduction utilizing a predictive regulator method was raised around (21.3-tonne) 13.6%, whereas 20.3% in the successive ten days simulation. It was concluded that the implementation of the solar collector, as well as the parabolic trough, worked coal-fired energy generation method has enhanced the understanding of the advantages and the restrictions of employing the method of a predictive regulator in the operating procedure. The choice of condenser cooling most likely has an impact on the techno-economic feasibility. In this regard, an effort is being made to assess the lifetime of CO2-eq pollution reduction (LCCM) capability for (50 MW) minimum capacity of both dry and wet cooling. Aseri et al.^3^ Carried out this research in India using two regions (PTSC) as well as (SPT) dry cooling dependent CSP facilities (6.0 h) of heat energy stored. The results showed that dry cooling may save a large quantity of water by 91.99% in these facilities corresponding to wet cooling facilities. Wang et al.^21^ suggested, fabricated, and experimented with a unique parabolic channel solar receiver with a radiation shield, according to the approach of a negative thermal flux zone, to improve the solar /thermal convert performance of the operational channel collector after its degradation at the maximum operational temperatures. They established mathematical patterns of heat collecting, as well as economic evaluation. The results of the simulation deliver good accordance with practical data. The techno-economic attainments of the solar power plants establishing the presented solar receivers in three regions underneath various installed capacities and thermal storage capacities were exhaustively studied. The findings pointed out that the presented solar receiver possesses a remarkable possibility for important improvement of the techno-economic achievement of the solar power system. Where the improvement of the yearly net electrical power output of the solar power system with the presented solar receivers placed in Dunhuang is around 9.77%, and a decrease in the Levelized cost of power is around 8.67%. Manesh et al.^22^ performed the development of a shared power plant in Qom city, which was started on the basis of a solar energy multi-impact desalination process. Considering this, they conducted a (6E) examination of energy, exergy, exert economic, exergoenvironmental, emerging economic, and emergoenvironmental. In addition, they used a multi-objective genetic algorithm (MOGA) to refine the proposed cycle based on the (6 E) analysis. The results showed that the suggested plant's exegetic performance improved by 3.22%. Furthermore, following optimization and at the best operating states, energy generation prices, the environmental effects of power generation, freshwater generation prices, the environmental effects of freshwater production, and the energy of the proposed system decreased by approximately 6.27%, 24.51%, 36.51%, 26.13%, and 1.87%, respectively. Linrui et al.^4^ created a model of a parabolic trough power plant and investigated its operating strategy. There is a sun field as well as a streamlined power block. They demonstrated that the adopted technique enhanced electrical power generation by 3.4 percent when compared to the original strategy. Wei et al.^23^ developed a novel dynamic analysis pattern for heat exchanger locomotives. Further, a simple analytical pattern of a combined parabolic trough CSP comprising a PTS part, a subsystem of energy-mass, and heat power storage is suggested for the first time. Validation between current platform data versus calculated data by the Andasol II facility occurred to demonstrate the accuracy of the integrated pattern. Each validation result of a steady-case and a couple of dynamic scenarios shows that the presented pattern can describe major system operations with acceptable accuracy and computing performance. Given the benefits of dependability and clarity, the integrated pattern can be used to develop and evaluate system controls for CSP facilities. ASI systems provide DNI data for the entire plant at a resolution of (20 × 20 m^2^), whereas the shadow camera system provides DNI data at a resolution of (5 × 5 m^2^). Both methods track cloud motions and, as a result, provide short-term forecasts of up to 30 min. These forecasts are used for sophisticated regulation methods in the solar field, potentially increasing the plant's overall yield by up to 2%^24^. Liu et al.^9^ presented a SAPG system that preheats feed water using a parabolic trough and heats steam with a solar tower. The system performance under three distinct loads (100%, 75%, and 50%), as well as the normal hourly performance throughout four typical days, were examined. It can result in more than (10%) more increased solar exergy. Arslan et al.^25^ investigated the lower solar region in the Rankine loop. they assessed several parameters such as R600a, toluene, and cyclopentane. Also, they designed a plant that involves a solar domain, a sub-plant of thermal power storage, and an energy block for 24 h operating period free of the external power source. It found that the traditional loops have a better design with a net ratio of 0.0009012 billion US$ and they determined the best temperature and pressure of the turbine input as 380 °C and 3.25 bar, respectively. The parabolic trough based on a solar plant depends on reducing its Levelized Cost of Electricity, due to the direct recirculation of molten salt. This study emphasizes the changes and concerns that are relevant to the replacement of thermic oils with molten salts such as the heat-transmitting coefficient, pressure declining, resistance-freezing solutions, energy bloc design, and price. The results demonstrated pressure declines in the solar domain are shorter running molten salts rather than thermic oil due to elevated temperature operating ranges^12^. Rao et al.^26^ created a unique thermodynamics prototype to replicate the reaction behaviours of basic and regeneration of CO_2_-TRC-based trough CSP techniques in the presence of diverse fog disturbances. The results demonstrate that when the system's performance is examined, the cloud thickness has the most influence on the range of abilities, while the overcast length has the greatest influence on the recovery time. At the same fog formation, the regenerative system's recovery process might be three times that of the simple system. When subjected to the same cloud cover period, the simple system reached a steady state in less time. Many similarities exist between LFC and PTC in terms of their possible integration in an AT-based ICST profiled technique. Both methods are scalable to various sizes with no discernible scale impact in terms of cost and substances. Scalability is typically achieved in both circumstances by adjusting the aperture region as well as the distance of the straight receiver^27^. The operation of the parabolic trough solar generation system was modeled and enhanced by Wang^28^ under cloudy circumstances. The difference between the exergy performance of the consuming power and thermal storage and the thermal power systems was informed by foggy circumstances. The model data versus well-known trial data was validated. The Combined Energy-Exergy-Control (CEEC) behaviour was utilized in this study to consider the problem of developing an efficient thermodynamic system with adequate regulation features. For that purpose, an energetic and exergetic study for the submitted cycle was done, followed by accurate modelling of the parabolic trough collectors (PTCs). They illustrated the governing control equations and calculated the regulation system's reaction period consequently. The CEEC optimum strategy is provided by utilizing multi-target optimization to optimize energy/exergy performance while reducing the suggested cycle's settling time. The results showed a 36.06% progress in entire cycle energy performance and a 25.09% settling time. While the energy, exergy, and settling times showed 34.02, 28.25, and 17.63% progress in goal operation, respectively^29^. To compensate for the end loss, Reddy and Ananthsornaraj^30^ proposed a parabolic trough solar collector (PTC) with an extended absorber tube length. The trough length was 4.6 m, the trough width was 5.7 m, the focal length was 1.7 m, and the rim angle was 80.3°. This compensatory technique is effective for sizable trough collectors since the thermal dissipation percentage of the unheated region of the receiver was minimal in comparison to the overall system's heat-collecting efficiency. El Kouche and Gallego^31^ developed the numerical simulations of a PTC with temperature based on physical features. Mathematical expressions were created. Several known and recent correlations for heat transfer factors were modelled. Further, several numerical simulations that provided useful feedback on the progress and efficacy of the PTC plant in the chosen area were performed. Moreno et al.^32^ suggested employing synthetic neural nets to estimate the best flow rate provided by a regulator design to substantially reduce the computational load to 3 percent of the MPC calculation time. The neural networks were trained on a 1-month test dataset of an MPC-controlled collector field. The use of a variable number of measures as net inputs have been investigated. The results revealed that neural net regulates offer about the same mean power as MPC regulators, with variances of less than 0.02 kW as with most neural nets, less abrupt changes in output, and minor breaches of the constraints. Furthermore, the suggested neural networks function effectively even when using small multiple sensors and estimates, with the set of neural net inputs reduced to 10 percentage points of the actual size. In this competition, more recent CSP facilities utilize Molten Salts (MS) in the solar collectors as heat storage means and, as a Heat Transfer Fluid (HTF) in some circumstances. In the implementation CoMETHy, a molten salt heated membrane reformer combined with a pre-reformer was designed and extensively proven at the industrial level (up to 3 Nm^3^/h H_2_ permeate production) in a molten salt cycle^33^. Goyal and Reddy^34^ created a numerical thermal pattern to evaluate the performance of s-CO2 as an HTF in a solar PTC. They calculated the entropy induced within HTF by finite temperature variations and fluid flow friction using regional temperature and velocity fields. Further, they utilized an optical analysis method based on Monte Carlo Ray tracing. The results demonstrated reducing entropy created in the PTC receiver to a minimum at the perfect Reynolds number for each of the HTF's operating pressures and intake temperatures. The Bejan number calculates the contribution of entropy developed by heat transfer irreversibility to entropy developed by heat transfer and fluid flow irreversibility, where was amidst of (0.2–0.4) at maximum flow rates and near to 1.00 at minimum flow rates^35^. According to the nonhomogeneous temperature diffusion in the PTC cycle, there is a new approach to cascadingly involving several solar chosen absorbing paintings in various divisions of the storage cycle. To put the intended technique into action, two systems were considered: the multi-division approach and the ideal approach. It found that the multi-division and ideal approach produces higher efficiency than a traditional approach. Further, under the working temperature between 290 and 550 °C, the heat loss of the multi-division approach was decreased by 29%, also the thermal performance was improved by 4%^35^. Subramanya et al.^36^ studied experimentally the performance of the PTC, operating a rotational receiver tube by velocity from 0 to 4 rpm, various inner temperatures, and flow rates. Multiple parameters are examined, such as thermal performance, temperature boost, and friction characteristics. Findings revealed that the friction characteristic rises rapidly, besides increasing temperature difference values because of usage of the rotational receiver tube. The best improvement of the thermal performance caused by declining the inner temperature and raising the flow rate was 190.3% compared with the fixed receive tube. Stutzle et al.^37^ modelled a linear regulator to develop a 30 MWe SEGS VI PTPP to provide a regulation algorithm for approximating the behaviour of an operator. Regulator response is assessed across both a winter day and a summer day. The effect of the regulator with respect to the total output of the PTPP is also investigated. Little enhancement of the total designed PTPP output is obtained by collector outlet temperature regulation. Valenzuela et al.^38^. described a PTPP operating in a once-through mode utilizing feedforward and PI regulators during clear days and short-term variations in DNI. For this purpose, a configuration with partial use of conventional regulators was selected since PTPP operators have experience using this kind of regulator adjusting regulator settings according to various situations affecting PTPP dynamics and regulator performance, such as changes in PTPP design or modifications made to the system over time. In steady-state mode, the findings indicate that it is possible to keep all set points also during short-term transients of DNI. While In the event of extended periods of DNI gradients, it is hard to keep the steam temperature. Camacho et al.^39^ reviewed several auto-control technologies employed before 2007 to regulate the outlet temperature of SF with dispersed collectors. A categorization of both modelling and regulation concepts has been presented to illustrate the most important characteristics associated with the various approaches. Felhoff et al.^40^ developed two main kinds of unsteady models based on direct steam generation (DSG) in the PTPP. First, a discretized finite element model (DFEM) was developed to provide a more detailed description of the PTPP characteristics and an explanation of the PTPP's behaviour. In addition, a second movable-boundaries model (MBM) combining lumped inputs and dispersed data can be applied to predict the behaviour of the PTPP. A comparison of both models with actual results is given, with variations for different system parameters. The response to local perturbations inside the evaporation pathway is shown not to be well replicated by the MBM. However, the MBM provides significant calculation benefits if the irradiance on the entire SF is assumed to be identical. DFEM is recommended for analyzing local influences, deriving transfer functions or providing a deeper understanding of the system properties. Biencinto et al.^41^ implemented a quasi-dynamic model by TRNSYS environmental software of a 38.5 MW PTPP with DSG using the recommended approaches and compared the annual power output. According to the findings presented in that analysis, it was found that applying a sliding-pressure approach for steam pressure control in the PTPP with DSG was more beneficial than the fixed-pressure approach regarding net power generation. Biencinto et al.^42^ described an innovative design for a PTPP involving wide aperture collectors where CO2 in a supercritical state (sCO2) is chosen as the operating medium, and molten salt is used as the thermal storage fluid. In addition, a module-based construction of the solar field is presented, which decreases the need for blowers and heat exchangers while minimizing the hydraulic loop of the molten salt. A comparison of the anticipated annual performance of the novel approach is made with a reference PTPP performance which uses thermal oil as the HTF in the SF. Two simulation models are designed in the TRNSYS software environment to replicate the behaviour of both the new and the reference PTPPs. According to the findings of this work, the new PTPP design has the ability to provide an enhancement in the annual efficiencies by approximately 0.5% and reduce power costs by about 6% compared with the reference PTPP.

For improving the performance of PTPP, a dynamic simulator can be a high-performance tool to analyse the plants' parameters in terms of inputs, process operation, gaseous outputs, or cost-effectiveness.

Many different commonly available programs have been utilized for PTPP modelling, such as TRNSYS, DYMOLA, EBSILON Professional, etc. Recent use has also been made of APROS software for dynamic modelling and simulation of PTPP, as illustrated in^5, 43^. To understand the response of the PTPP towards meteorological conditions variations, such dynamic models are implemented. Evidently, only limited dynamic models have been introduced so far regarding PT technology. But to date, most of these efforts addressed the TSS and SF models, as well as limited research papers, that presented the PB dynamic models.

### Goals

It was designed with a thorough dynamic model (named optimization 2) with APROS software. In this designed model, a three-part model (SF, TSS, and PB) is developed containing all regulation loops needed to regulate the variations encountered throughout the PTPP's operation. Subsequently, that designed model is evaluated against the referenced model (validated model) in^5^ by comparing it for highlighting the improvement level. The novelty of the optimizations carried out in Optimization 2 within this paper is described as follows:

- Development of a dual model for preheating the feedwater using the Feedwater/HTF model during the day period and the Feedwater/Steam model during the night period. This design represents the first of its type in this area.
- Implementation of the steam turbine model to operate with and without steam extractions. The benefit of the first case is to get rid of reliance on the fossil fuel system during the night period, while in the second case, it is operated to increase electrical power production in the daylight period.
- Increase of thermal energy absorbed, concentrated in the SF, through expansion to more loops.
- Extending the night operation of the PTPP based on an increase in the capacity of the TSS.
- Comparing the cost of the new PTPP according to the annual performance of the plant.

## Modelling

APROS is capable of building progressive regulation circuits. Dynamic simulations over several days can be continually performed. As a result, APROS is regarded as the superior tool for modelling several power plants, particularly during dynamic processing, for its high ability to provide steady performance, accuracy, and fast response during rapid changes in load. Some more comprehensive details on the solution procedure realized in APROS can be found, e.g., in^44^.

The finite-difference or finite-volume method is usually used to solve one-dimensional partial differential equations. These equations are discretized spatially and temporally and the nonlinear terms are then linearized. For spatial discretization (integration over the appropriate segment length), multiple discretization techniques are available, including the first-order upwind technique, the second-order central differentiation technique, and the quadratic-upwind interpolation. The implicit approach is typically applied for temporal discretization. Finally, it is possible to calculate the physical properties including enthalpy, pressure and velocity within the computational model based on the discretized conservation laws, the inlet and outlet flow parameters, as well as the thermodynamic characteristics. In accordance with the software APROS^43^, which has used the finite-volume method for solving the one-dimensional partial differential equations, the method of the solution involves^44^:

The conservation of energy, mass, and momentum laws are imposed on the control volumes.

Libraries of material properties are accessible in terms of related variables such as specific enthalpy, pressure and mass fraction.

It is possible to apply single-phase, mixed-phase, non-equilibrium separating-phase, turbulent, critical and laminar flows. Moreover, radiations, convections and diffusions and appropriate heat transfer relations can be used. Chemical interactions can be associated with the considered control volumes.

The models of regulation system elements including regulators, logic inputs and processes, and sequential automation blocks can be functionally involved in the simulation model.

Experimental correlations are applied for the valid scope.

Electrical components can be added to the simulation model in a functional way, such as generators, electric motors, etc.

In the case of unsteady flows, discretization should be done in time, taking into account pertinent condition quantities and chosen time scales.

Determine the physical quantities of the operating medium within a control volume, including energy flow, mass flow and separating media.

## Power plant operating with a dual feedwater circuit (optimization 2)

**Figure 1:**
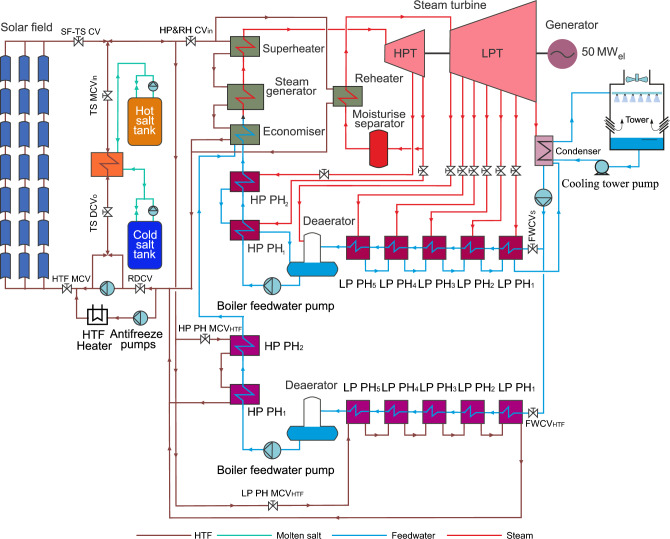
Schematic diagram of a PTPP with dual feedwater circuit.

In order to achieve high flexibility during the operating periods for the PTPP, modifications were made to the optimized feedwater and steam turbine models. Furthermore, the specifications of the reference power plant and optimization 2 are listed in Table 1. This development is called optimization 2, as illustrated in Fig. 1. In the following sections, a dual feedwater circuit and steam turbine circuit as well as the regulation circuits for these optimized circuits will be described.

**Table 1:** Specifications of the reference power plant and optimization 2.

Specifications	Reference power plant	Optimization 2
Number of loops	156 loops	208 loops
Number of collectors per loop	4 collectors	4 collectors
Type of collector	Parabolic trough	Parabolic trough
Storage capacity	28,500 tons of molten salt (1025 MW_th_ h)	38,000 tons of molten salt (1360 MW_th_ h)
Heat transfer fluid	Therminol VP-1	Therminol VP-1

### Dual feedwater circuit 

The optimized feedwater circuit implemented in optimization 1 is developed using APROS software. The referenced feedwater/steam circuit (FW/S circuit) is combined with the feedwater/HTF circuit (FW/HTF circuit) improved in^45^. The evening period can be divided into two parts based on the electricity demand, namely high and low demand. The high demand period begins when the sunset and continues until approximately 10:00 pm, while the low demand period starts from 10:00 pm until 6:00 am. The objective of this design is to enhance the operation hours of the evening period by reducing the nominal power from 61.93 to 48 MW_el_. This will provide operational flexibility and steadier electricity generation when the demand for energy is low during the evening period. Furthermore, this optimization decreases the dependence on fossil fuel backup system during the evening hours.

**Figure 2:**
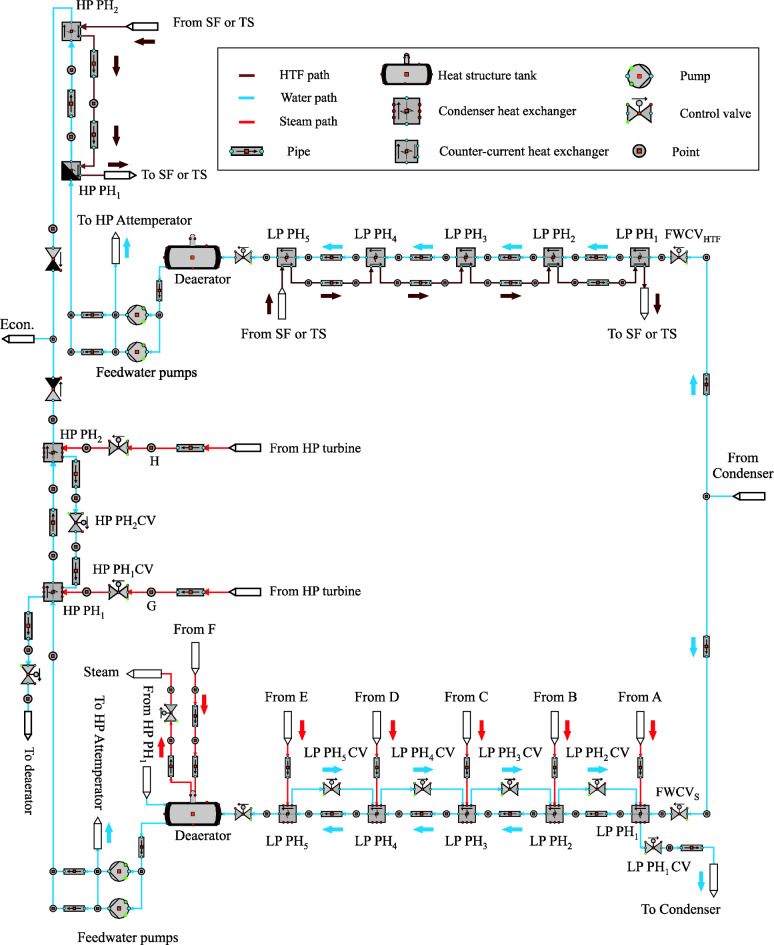
Dual-circuit feedwater model.

Dual-circuit feedwater is modelled using two trains of heat exchangers (Train 1 and Train 2), where each heat exchanger is considered a preheater, as depicted in Fig. 2. In train 1, five counter-current heat exchangers at low-pressure feedwater are connected together to the deaerator. The feedwater after the deaerator is pumped into two counter-current heat exchangers at high-pressure feedwater. The HTF flows through the heat exchangers of train 1 to heat the feedwater passed through the tube side of the FW/HTF circuit during the daylight hours. Train 2 includes seven condenser heat exchangers similar to the reference feedwater circuit, where five heat exchangers are used as the LP preheaters and two of them as the HP preheaters. The steam extracted from the turbine is used to heat the feedwater passed through the second feedwater circuit (FW/S circuit). It is worth mentioning that a certain amount of the feedwater is pumped to the high-pressure attemperator either from FW/HTF circuit or from the FW/S circuit or from both circuits in order to adjust the temperature of bypassed steam from the HP bypass control valve before entering into the reheater.

The FW/HTF circuit is operated during the daylight, while the FW/S circuit is used in the evening. During the sunset period (transient period), an economizer uses feedwater from both feedwater circuits with a different amount. Here, the boundary conditions of feedwater after and before each preheater in both circuits are maintained at similar values to those predefined in the reference circuit. Furthermore, the boundary conditions values of steam and HTF used in both circuits are kept similar to the reference and optimized feedwater circuits.

#### Dual-circuit feedwater regulator loops

All regulator circuits implemented in the reference and the optimized feedwater circuits are used in dual-circuit feedwater with the same boundary conditions, except for FW MCV_LP_ in both circuits. However, the regulation structures of FW MCV_LP_ are changed in both trains, where two control valves are added before the heat exchangers train, namely the feedwater control valve before the FW/HTF circuit (FWCV_HTF_) and ahead of the FW/S circuit (FWCV_s_). Furthermore, three regulation circuits in the boiler and thermal storage system are modified. The operation mode of new control valves will be described in the following sections.

##### Feedwater regulator in FW/HTF circuit (FWCV_HTF_)

**Figure 3:**
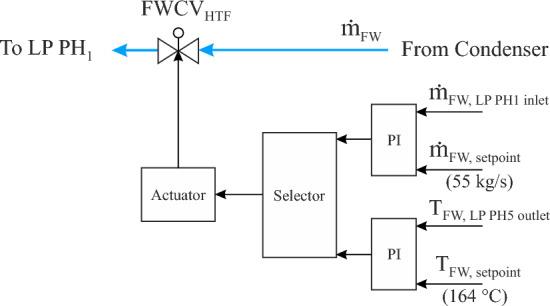
Feedwater regulator in the FW/HTF circuit (FWCV_HTF_).

The feedwater regulation valve is located ahead of the first LP preheater (LP PH_1_) in the FW/HTF circuit. Two tasks are implemented in this regulation circuit depending on the operation mode of the power plant, as shown in Fig. 3. This regulation circuit includes a single selector which in turn changes between these tasks based on two boundary conditions (the HTF mass flow and temperature at the SF outlet must be less than 802 kg/s and 393 °C, respectively, as well as when the transient period should be started). The transient period starts when the HTF mass flow at the SF outlet decreases to less than the nominal value of 802 kg/s due to the sunset period. The first task is activated after sunrise, where the FWCV_HTF_ is gradually opened until achieving the basic quantity of 55 kg/s within the FW/HTF circuit and it remained unaltered until the transient period. The second task is operated when both boundary conditions are achieved. Therefore, the FWCV_HTF_ maintains the HTF temperature at the LP PH_5_ outlet unaltered at the design temperature of 164 °C by regulating the feedwater mass flow passed through FWCV_HTF_. In the transient period, a part of the hot feedwater is supplied by the FW/HTF circuit and the rest is provided through the FW/S circuit with the same properties (temperature and pressure). As a sequence, the FWCV_HTF_ will totally be closed when the HTF mass flow at the SF outlet is equal to zero. In the evening period, the FW will be supplied from the condenser through FWCV_S_, as explained in the next section.

##### Feedwater regulator in FW/S circuit (FWCV_S_)

This regulation valve is installed at the inlet of the FW/S circuit. The purpose of FWCV_S_ is to regulate the feedwater mass flow through the FW/S circuit during the evening period and in the transient period. The operation mode of FWCV_S_ can be described as follows.

**Figure 4:**
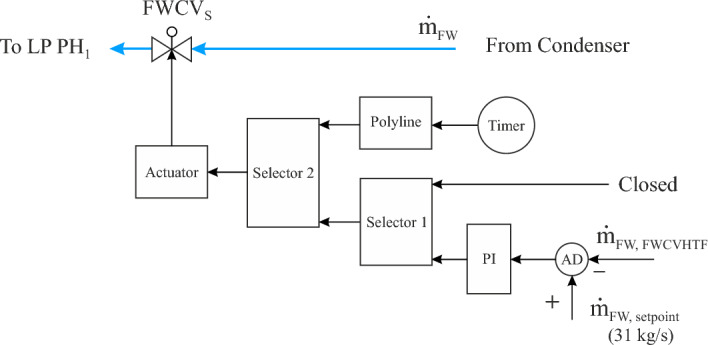
Feedwater regulator in the FW/S circuit (FWCV_S_).

The mass flow of feedwater after the FWCVHTF is recorded and then compared to the setpoint (31 kg/s). This comparison is implemented by a comparator (AD) which in turn sends a signal to the PI controller, as depicted in Fig. 4. Thereafter, a PI controller commands the actuator which operates FWCV_S_. This control valve achieves two functions depending on two selectors. The first selector includes one boundary condition (feedwater mass flow after the FWCV_HTF_ must be less than 44 kg/s), while the second selector also consists of a single condition (the HTF temperature at the TS outlet must be less than 377 °C). The first function is activated during the solar mode of the power plant, where this regulation valve remains closed until the transient period. After accomplishing the condition of selector 1, the second function of this selector is operated, where the rest of the feedwater mass flow passed into the economizer will be replenished through FWCV_S_ depending on the thermal storage system. When the HTF temperature at the thermal storage outlet falls below 377 °C, the FWCV_S_ will be closed gradually by means of a time gradient (polyline).

It can be noticed that the path change between train 1 and train 2 occurs in the transient period (before sunset) by opening the FWCV_s_ and closing the FWCV_HTF_, gradually.

##### HTF regulator at the superheater and reheater inlets 

**Figure 5:**
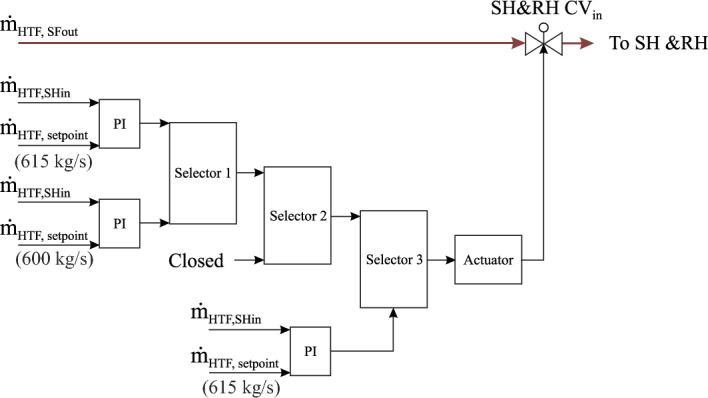
HTF regulator at the superheater and reheater inlets.

The HTF regulation valve at the power block inlet adjusts the HTF mass flow rate, which enters the superheaters and reheaters. In this regulation circuit, there are three selectors. Two functions are passed through a selector. In selector 1, the first task is operated when the sun rises, where this valve maintains the HTF mass flow at the superheaters and reheaters inlet at 615 kg/s according to the predefined setpoint. Thereafter, it continues regulating this value until the transient period. The second task is activated after accomplishing two boundary conditions (the HTF mass flow at the SF outlet must be less than 802 kg/s and the transient period should be started). This process is obtained by comparing the HTF mass flow rate at the superheater and reheater inlets with a new setpoint of 600 kg/s through the PI controller, as shown in Fig. 5. In the second selector, two tasks will be passed through selector 2. The first task comes from selector 1 and the second one is activated when the level in the hot storage tank falls below 0.6 m and there is no HTF coming from the solar field outlet, where this valve will be closed. In the third selector, the first function is received from selector 2 and the second function is operated based on two boundary conditions (the HTF mass flow at the SF outlet must be more than 390 kg/s and the temperature more than 295 °C). When both conditions are achieved, this valve regulates the mass flow of HTF in the superheater and reheater inputs at 615 kg/s according to a setpoint.

##### LP and HP main regulators of HTF at the optimized feedwater circuit

**Figure 6:**
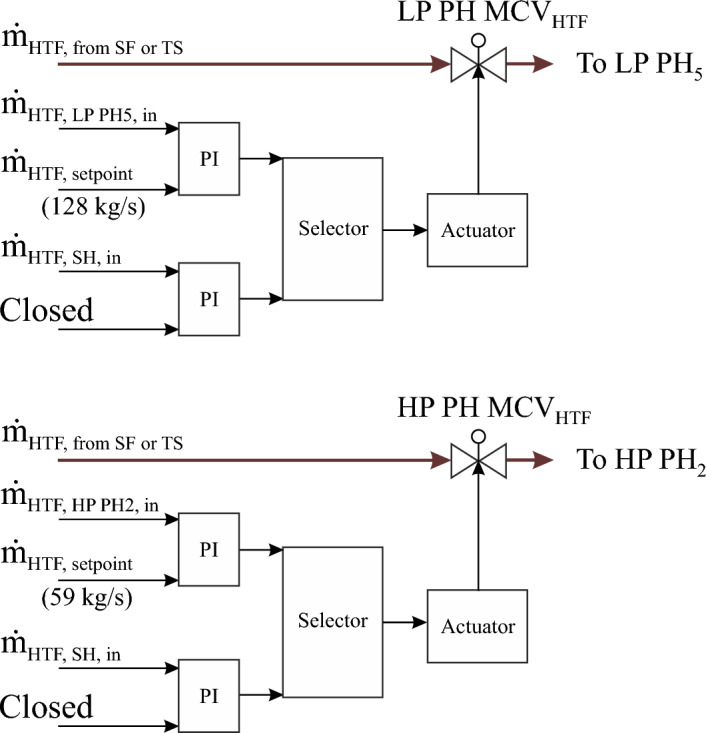
LP and HP main regulators of HTF before preheaters.

The regulation structure of LP and HP main control valves of HTF before the optimized HP-preheaters and the LP-preheaters (LP-PH MCV_HTF_ and HP-PH MCV_HTF_) are improved, as demonstrated in Fig. 6. Both control valves apply the same working principle with varying constraints in order to adjust the mass flow of HTF, which is sent for each type of preheater. These regulation circuits include two selectors for each one. Two functions are passed through each selector. The functions in the selectors used for both circuits are changed depending on the same boundary conditions (the HTF mass flow at the SF outlet must be less than 802 kg/s and the transient period). The first function is activated after sunrise until the transient period, with the LP PH MCV_HTF_ and the HP PH MCV_HTF_ controlling the HTF mass flow rate at inlets LP-PH5 and HP-PH2 using setpoints equal to 128 kg/s and 59 kg/s for the LP and HP preheaters, respectively. After achieving both conditions, the second function is operated, where both regulators maintain the mass flow of HTF at the superheater inlet at 600 kg/s until the thermal storage energy is depleted. The LP PH MCV_HTF_ and HP PH MCV_HTF_ are fully closed when the nominal HTF mass flow at the superheater and reheater inlets (600 kg/s) is reached. After depletion of the stored energy, the transient time of the next day must be initiated in order to repeat the same approach of these regulation circuits.

##### Optimized HTF regulators at the improved storage system outlet (TS DCV_o_)

**Figure 7:**
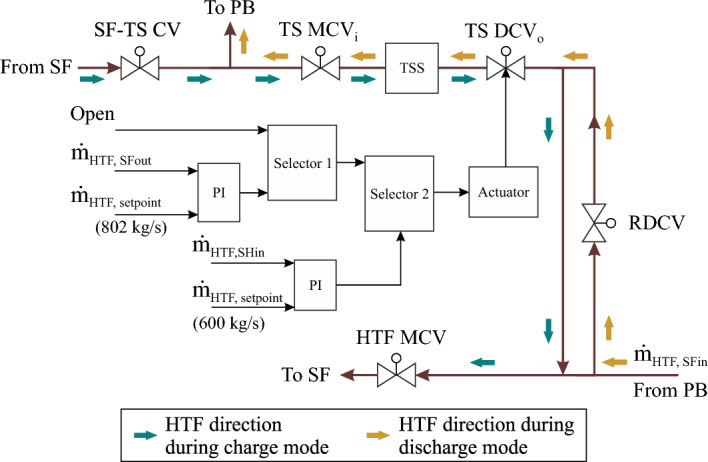
HTF regulator at the thermal storage outlet in the dual circuit.

The regulation valve at the optimized TSS outlet is improved by adding a new selector as well as changing the setpoint of HTF mass flow and boundary conditions, as displayed in Fig. 7. In the compensation period, the TS DCV_o_ can be considered as the inlet of the thermal storage system, where it regulates the HTF mass flow which enters the optimized TSS to accomplish the nominal value of 802 kg/s in the day and 600 kg/s in the evening. The operation strategy of the TS DCV_o_ can be described below:

This regulation circuit includes two selectors, i.e. two functions are passed through each one based on the operation mode of a storage system. The first function is operated during charge mode, where it remains open in order to allow the cold HTF at a temperature of 293 °C to flow from the TSS into the solar field. The second function is activated after achieving these conditions (either the HTF mass flow at the SF outlet is less than 802 kg/s or its temperature is less than 393 °C, as well as the level of the hot storage tank, must be more than 0.6 m). Accordingly, the HTF mass flow at the SF outlet is measured and compared to the setpoint (802 kg/s) by a PI controller. As a result, the second selector depends on two boundary conditions (the transient period should be started and the level of the hot storage tank is more than 0.6 m), where this control valve will replenish the HTF mass flow according to the new setpoint (600 kg/s).

It should be mentioned here that the same procedure used in TS DCV_o_ is applied to the redirection control valve (RDCV).

#### Steam turbine model with extraction regulators

**Figure 8:**
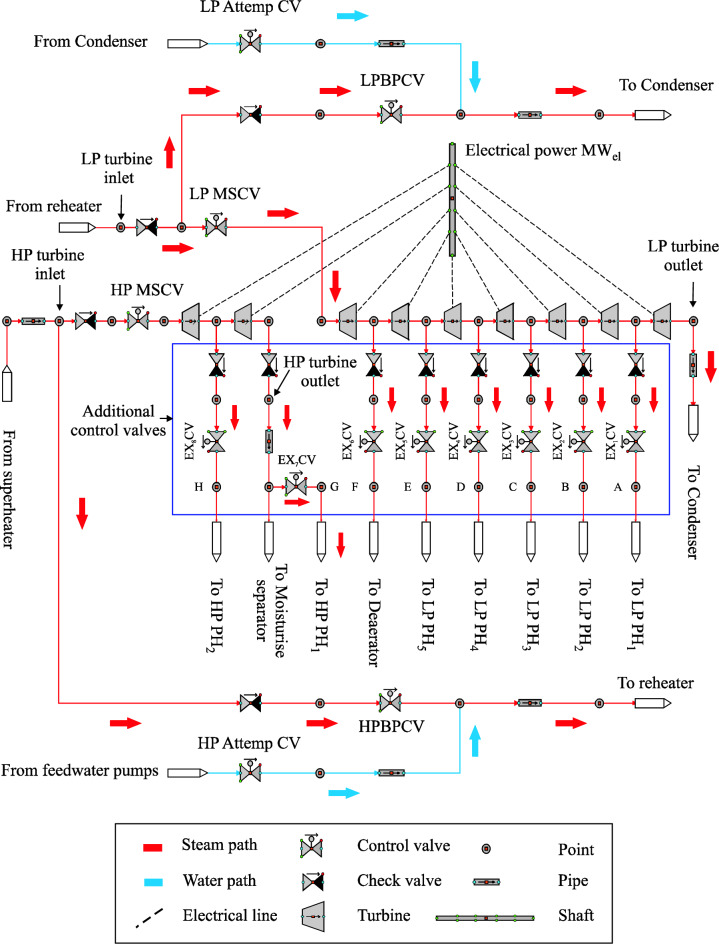
Steam turbine model with extractions regulation valves.

The reference steam turbine (ST) is developed by adding eight control valves for the steam extractions, two of them are connected to the HP preheaters and six valves are connected to the LP preheaters, as depicted in Fig. 8. During daylight hours, the steam turbine is operated similarly to the procedure followed in optimization 1, while these regulation valves remain closed until the transient period. Therefore, 55 kg/s of steam enters the HP-ST and leaves LP-ST as well as the pressure and temperature, which are maintained similarly to the optimized model. It is worth mentioning that the economizer only obtained the feedwater supplied through the FW/HTF circuit in the daytime period. Thereafter, the regulation valves of steam extractions are opened gradually in the transient period until achieving the design mass flow of steam extractions. During the transient period, the economizer received the nominal value of feedwater from the FW/HTF circuit and FW/S circuit. After sunset, the entire nominal value of feedwater (49 kg/s) is provided to the economizer by the FW/S circuit. It should be mentioned here that all properties of steam and feedwater (mass flow rate, pressure and temperature) during the day are set similarly to the optimized model and in the evening similar to the reference model.

All regulators modelled in the reference steam turbine model are used in this optimized steam turbine model. A noticeable difference can be seen in the LP MSCV circuit, which regulates a flow rate of steam mass at the inlet of the LP-Turbine to 55 kg/s during the day instead of 46 kg/s, while it remains at the same design value (41 kg/s) at the night period. This will provide more operating hours in the evening. Furthermore, the extraction regulation valves are modelled in order to operate the steam turbine with a high level of flexibility and accuracy in all operation periods. The working principle for these regulation valves is explained in the following.

##### Steam extraction regulation valves (EX CV)

**Figure 9:**
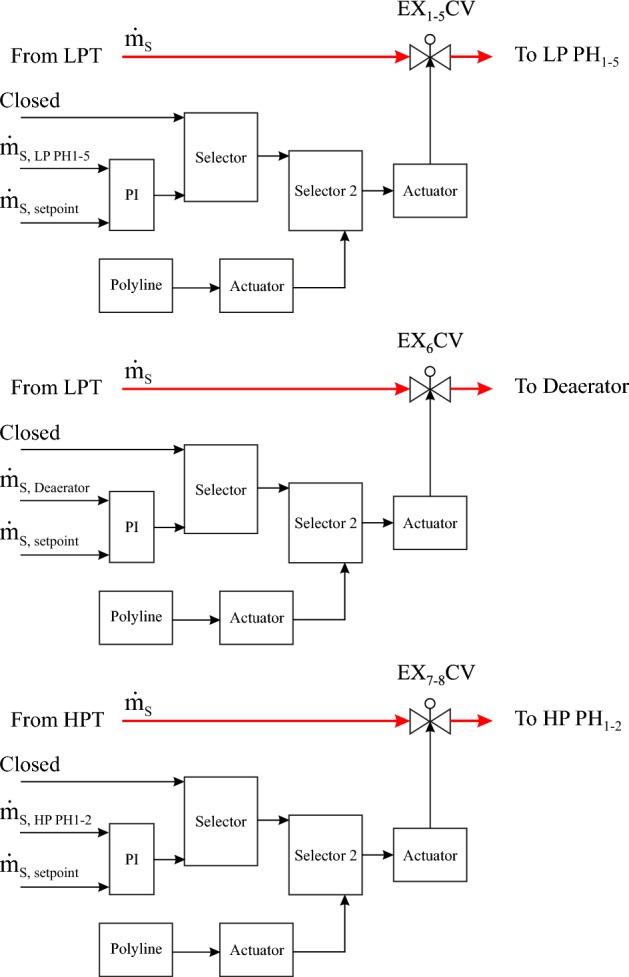
Steam extraction regulators.

There are eight steam extraction regulation circuits in the steam turbine model, as described in Fig. 9. The regulation structures and operation strategies are the same for all steam extraction regulation valves but with different mass flow rate setpoints and locations. The steam extraction regulation valves from 1 to 5 are connected to the LP PH_1_, LP PH_2_, LP PH_3_, LP PH_4_ and LP PH_5_, respectively. The sixth steam extraction regulation valve is installed between the sixth extraction and the deaerator, while the seventh and the eighth regulation valves are connected to the HP PH_1_ and HP PH_2_, respectively. The operation mode of steam extraction regulators will be explained as follows:

Each regulation circuit includes two selectors, where two tasks are passed through a selector. In the first selector, the steam extraction regulation valves are remained closed as long as the boundary condition (the designed value of feedwater mass flow in the FW/HTF circuit is equal to or more than 44 kg/s) is still not accomplished. Subsequently, the second task is activated after accomplishing this condition, where the steam mass flow is regulated according to the setpoints. Thereafter, these regulation valves continue maintaining the design extraction values as long as the HTF temperature at the TS MCV_i_ during discharge mode is equal to or more than 377 °C. Two functions are passed through the second selector based on one boundary condition (the HTF temperature at the TS MCV_i_ during discharge mode must be less than 377 °C). The first function is received from selector 1 as long as the boundary condition in selector 2 is not yet achieved. On the other hand, these regulation valves are gradually closed according to a time gradient (polyline) when the boundary condition in selector 2 is upheld. Thereafter, they will remain closed until the transient period of the next day.

## Results

The second optimization of PTPP will be summarized and discussed throughout the clear days, together with a thorough assessment of the PTPP model in this section. The dual-circuit feedwater in the PTPP is operated using two trains of heat exchangers (Train 1 and Train 2). Train 1 uses thermal oil to keep the feedwater passing by HTF/FW circuit hot throughout the daylight hours. In the evening period, train 2 is used instead of train 1. Train 2 is operated using the steam extracted from the turbine to heat the water passed through the second feedwater circuit. This change between train 1 and train 2 is implemented in the transient period (before sunset) by opening the FWCV_s_ and closing the FWCV_HTF_ (see “Dual-circuit feedwater regulator loops”). The aim of this process is to increase the operating hours during the night period by decreasing the nominal load from 61.93 to 48 MW_el_ because of low energy consumption during the evening period. Furthermore, this optimization reduces dependence on the fossil fuel backup system during the night hours.

In the following sections, several comparisons between the validated PTPP (reference) on clear Summer days and the improved PTPP (optimization 2) are presented and discussed.

### Optimized HTF behaviour in the solar field 

A comparative analysis between the improved PTPP (Optimization 2) and the validated PTPP (Reference) is performed in this section. This comparison is achieved according to the predefined definitions in ref. ^5^. The predictions obtained are assessed separately considering a description of the various properties of each section of the PTPP. It should be mentioned here that the limitations of the optimized total mass flow rate in optimization 2 are similar to those in optimization 1. Hence, the same heat collected in optimization 1 is applied to the solar field in optimization 2. Reference model predictions and optimized outputs are analyzed for 26th–27th/June and 13th–14th/July 2010, along with the total HTF mass flow and temperature at the power block. A discussion regarding each of these properties is provided in the following subsections.

#### HTF mass flow rate in the SF (optimized)

**Figure 10:**
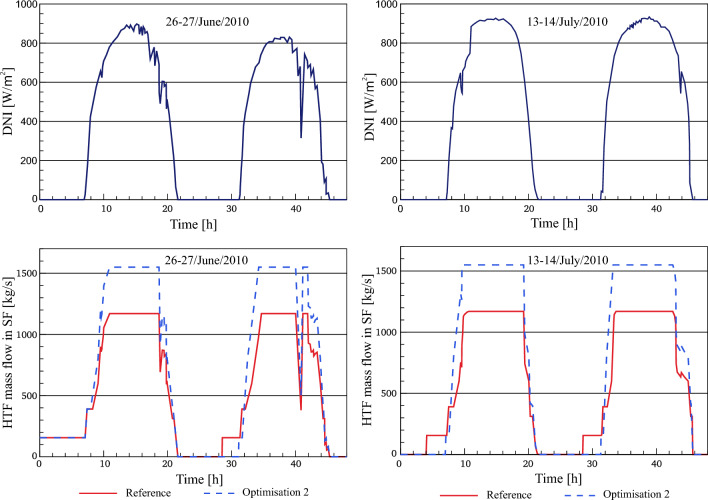
Description of total HTF mass flow.

A comparison between the referenced and improved total mass flow is presented across the representative days, as displayed in Fig. 10. In the period between t = 00:00 and t = 7:30 on 26th/June/2010, the optimized total mass flow rate matches with the reference plant. The reason for this situation is that the end of the thermal storage period is not available from the operating company. Therefore, the optimized total mass flow rate for this period is assumed to be like the optimized results in optimization 1. This, in turn, leads to similar results for all properties until sunset compared to those obtained from optimization 1. According to optimization 2, the total HTF mass is not circulated in the optimized solar field for additional hours, around (2.5–3 h) more than the reference solar field due to the stored energy is still not depleted. According to the operation strategy implemented in the power plant, HTF cannot be circulated in the SF when the TSS is still being discharged. After depletion of the TSS, the optimized mass flow starts at a circulation rate through the SF with a constant value of 156 kg/s. Thereafter, a simple change in the operation strategy of optimization 2 is carried out, where the optimized total mass flow rate remains unaltered at 156 kg/s despite the sunrise. After reaching the design outlet temperature (393 °C), it increases to the designated value (802 kg/s).

In turn, this translates into fast startups, since the HTF's designed outlet temperature (393 °C) is achieved more quickly in optimization 2 versus the referenced model. In contrast, after sunrise, the referenced mass flow rate rises from 156 to 390 kg/s. Consequently, it starts increasing after accomplishing the design inlet temperature (295 °C).

#### HTF temperature at the outlet of the solar field (optimized)

**Figure 11:**
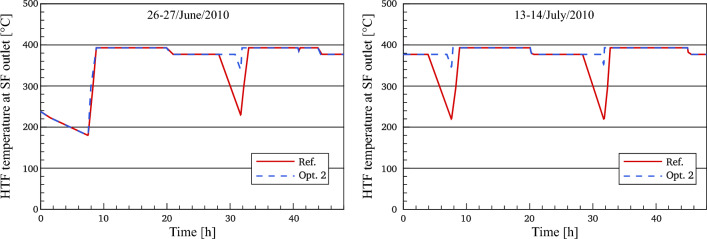
Description of HTF temperature.

The HTF temperature at the outlet of the solar field in Optimization 2 is analyzed against the referenced model, as illustrated in Fig. 11**.** As demonstrated previously in “HTF mass flow rate in the SF (optimized)”, the HTF temperature in optimization 2 and optimization 1 is the same due to the unknown strategy of thermal storage in the real power plant before sunrise on the 26th of June. During sunset periods for the rest of the selected days, the design outlet temperature of HTF decreases from 393 to 377 °C according to the operation strategy applied in the model. It can be noticed that the HTF temperature (377 °C) in optimization 2 is achieved in around 10 min less than the reference model. This is since the HTF mass flow used in the optimized power block during the evening period is 600 kg/s as the reference model but the collected heat during this period in optimization 2 is more than for the reference model. Subsequently, the HTF temperature continues unchanged for a period of time ranging between 155 and 196 min more than for the reference model. When the stored energy is fully depleted, the HTF temperature decreases by natural cooling. When the sun rises again, the HTF temperature starts increasing to the design outlet temperature of the SF (393 °C) by a period of time approximately one hour shorter than it is in the reference model. This improvement in the optimized HTF temperature results from several influences: a rise of the accumulated heat inside the optimized SF, the application of the accumulated heat to 156 kg/s instead of 390 kg/s and the short period of time between the sunrise and the depletion of thermal storage, i.e. the HTF temperature in optimization 2 does not have sufficient time to drop more than this value under natural cooling. Afterwards, the same operation strategy in optimization 2 is repeated for the next days.

### Optimized HTF behaviour in the thermal storage system 

A discussion of the optimized HTF behaviour of the TSS compared to the referenced model is given in the following sections. It should be mentioned here that the specifications of the optimized thermal storage system in optimization 2 are similar to those defined by optimization 1 in ref. ^45^. Hence, the same energy stored in optimization 1 is used in the TSS in optimization 2. However, the HTF mass flow to the TSS and the stored energy presented and analyzed here are compared to the reference model results.

#### HTF behaviour to the thermal storage system (optimized)

**Figure 12:**
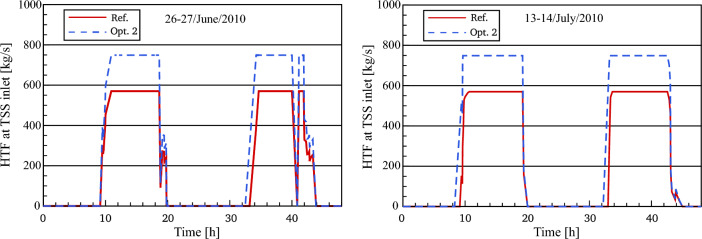
Description of HTF behaviour to TSS.

Figure 12 illustrates the difference between the referenced and optimized HTF mass flow to the thermal storage system for the chosen days. On 26th June, there is an observation that the behaviour of the HTF mass flow to the TSS in optimization 2 is similar to that presented in optimization 1. The reason for this is the unknown strategy of thermal storage in the real power plant before sunrise on the 26th of June. As a result, the optimized mass flow to the TSS for this period is assumed to be like the optimized predictions in optimization 1. For the rest of the selected days, the optimized HTF is found to begin flowing into the TSS earlier than the referenced mass flow, approximately 42–50 min. This leads to achieving the maximum value of HTF mass flow (749 kg/s) in optimization 2 faster than in the validated plant. Thereafter, it remains unaltered for an additional time of approximately 30 min longer than in the reference model. Generally, the HTF mass flow to the thermal storage system in optimization 2 approaches the same behaviour as in the referenced model until the end of the day.

### Optimized thermal storage energy

**Figure 13:**
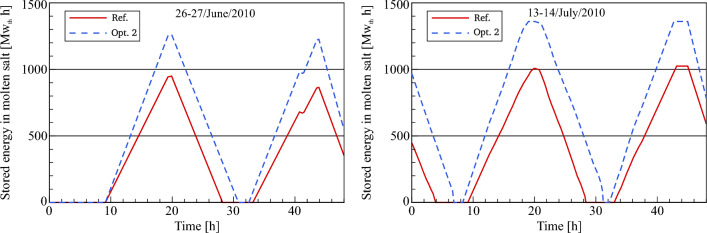
Description of storage energy behaviour.

Figure 13 shows the optimized accumulated stored energy compared to the validated model. As explained in Optimization 1, the thermal storage system is enhanced when its capacity is increased. Therefore, the same capacity of the TSS mentioned in optimization 1 will be used in optimization 2. It should be noted that the temperatures of the HTF at the inlet and outlet TSS are assumed to be similar to optimization 1. On 26th June, as explained in the previous sections, the referenced and optimized thermal storage started increasing at the same time. Due to the fact that the improvement in optimization 1 is not applied for this period, which precedes sunrise on the 26th of June. As a result, the heat storage increases until reaching the same value in optimization 1 (1260 MW_th_ h). For the rest of the selected days, the optimized accumulated storage energy begins increasing about 42–50 min before the referenced storage energy. This will provide enhancement in the stored energy, where on 27th June there is an increase in the maximum storage energy of around 78 MW_th_ h versus optimization 1. On 13th July the accumulated storage energy in the hot storage tank increases to reach the maximum capacity of 1360 MW_th_ h. In contrast to optimization 2, the maximum value of energy stored in the hot storage tank was not achieved in the reference model and optimization 1. While on the 14th of July, the maximum value of thermal storage (1360 MW_th_ h) in optimization 2 is reached faster than in optimization 1. During the sunset period, the optimized storage energy starts dropping at the same time as in optimization 1 and the reference plant, as illustrated in Fig. 13. It can be seen from this comparison that optimization 2 provides some additional hours for the night operating periods ranging between 155 and 179 min more than the reference model and optimization 1. This enhancement can be attributed to three reasons: an increase in the energy stored within the optimized TSS, the usage of the stored energy to heat 600 kg/s instead of 802 kg/s and the short period of time between the sunrise and the depletion of thermal storage, i.e. the design outlet HTF temperature in optimization 2 is reached faster during the daytime than in the reference model.

When the TSS is completely charged, as observed in the days of July, some collector rows are some rows of collectors are facing the ground preventing the HTF designated outlet temperature from rising above the limit (393 °C).

### Optimized HTF behaviour in the power block

A discussion of the optimized HTF behaviour in the power block compared to the reference model in ref. ^5^ is presented in the following sections. Here, the preheaters in the FW circuit are operated using two working fluids, where the thermal oil is used through the shell side for the first train of heat exchangers during daylight hours. At night, the steam extracted from the HP and LP turbine is used within the shell side for the second train of heat exchangers to heat the FW passed in the tube side. As a result, the HTF mass flows to the PB, the thermal power, and the total electrical power are evaluated against the validated results of the referenced model.

#### HTF mass flow rate to the PB (optimized)

**Figure 14:**
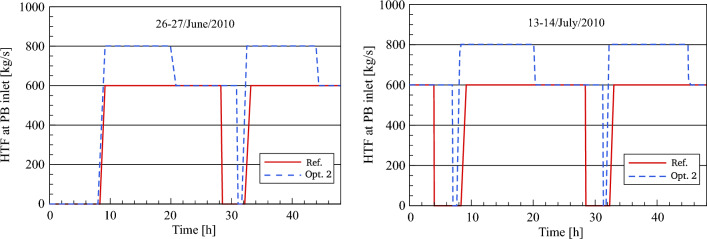
Description of HTF behaviour to the power block.

In Fig. 14, the HTF behaviour optimization 2 and reference model to the power block are presented and discussed for the chosen days. On 26th June, the resulting HTF mass flow to the PB in optimization 2 completely matches with the results in optimization 1 until sunset due to the reasons outlined earlier in the paper. For the rest of the chosen days, it can be observed here that the PB in optimization 2 starts receiving the HTF from the optimized solar field about 30–41 min earlier than the referenced model. Here, observe for each selected day that the HTF mass flow rate increases toward reaching its rated amount of 802 kg/s at 393 °C and remains unchanged until sunset. At the beginning of the transient period, the HTF mass flow to the power block is gradually reduced to 600 kg/s at a constant temperature of 377 °C. Thereafter, the nominal mass flow of HTF (600 kg/s) is kept unaltered for the evening periods for a period of about 10–10.5 h due to an increase in the stored energy. As a result, the HTF mass flow falls to zero. On the other hand, the HTF mass flow maintains constant at 600 kg/s throughout the day and night times until the depletion of stored energy.

The HTF mass flow in optimization 2 is found to remain unaltered at this value (0 kg/s) for a period of approximately 26–37 min before sunrise. In contrast to optimization 2, the HTF mass flow is not circulated through the PB in the reference model for a period of about 220–260 min before sunrise. After sunrise the next day, the same scenario in optimization 2 is repeated in the following days.

#### Thermal power to the PB (optimized)

**Figure 15:**
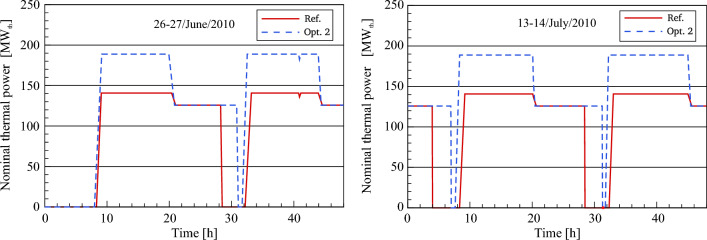
Description of thermal power to the power block.

A comparison between optimized and referenced thermal power is presented for the typical days. It should be noted that the heat absorbed in the SF of optimization 2 is the same amount in optimization 1 due to using the same solar field in both models. It can be noticed that the thermal power is transmitted to the power block in optimization 2 approximately 30–41 min ahead of the referenced model. The reason for this is that the HTF design inlet temperature (295 °C) is achieved faster than it is in the reference plant, as shown in Fig. 15. Thereafter, the thermal power in optimization 2 rises to the designated value of 188.78 MW_th_ and after that remains unaltered till sunset. During the sunset period (transient period), it falls from 188.78 to 125.75 MW_th_. While the thermal power is reduced from 140.72 to 125.75 MW_th_ in the reference plant. During this period, the thermal storage starts supplying the required thermal power to the PB together with the heat collected in the solar field. In the transient period, the regulation valves (HP and LP PH MCV_HTF_) are gradually closed and then it is totally closed when sunset. This leads to preventing the HTF from flowing into the FW/HTF circuit after sunset. Therefore, the entire incoming HTF (600 kg/s) is only sent to the steam generator. It can be seen here that the optimized thermal power remains unchanged at 125.75 MW_th_ for a period ranging between 2.5 and 3 h more than it is in the validated plant. When the TSS is completely depleted, the thermal power maintains at a constant value of 0 kg/s until achieving the design inlet temperature of HTF (295 °C). In this case, an observation can be made that the optimized thermal power is again transferred to the power block for a period of about 26–37 min before the reference model. Then, the same scenario is repeated for the next days.

#### Gross electrical power (optimized)

**Figure 16:**
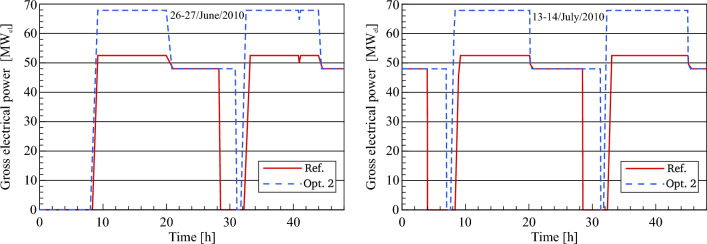
Description of gross electrical power.

The gross or total electrical power simulated by Optimization 2 is analyzed relative to the outputs predicted using the reference power plant along the chosen periods, as illustrated in Fig. 16. The purpose of this comparison is to explore the effect of optimization 2 on electric power produced by the thermal cycle. The power optimization and operation strategy have a significant impact on the enhancement of energy production. This enhancement is highly noticed in the optimized gross electrical power, where it increases to a maximum value of 68 MW_el_ in the daytime and 48 MW_el_ at nighttime. During the night period, the optimized power plant obviously produces electrical power at a constant value of 48 MW_el_ for a period of approximately 10–10.5 h. On the other hand, the electrical power in the reference model is produced at a constant value of 48 MW_el_ for a period of approximately 7.5 h. This is due to the fact that the storage capacity is increased and that the operation strategy of the power plant is improved for the daytime hours and the evening period.

### Optimized steam behaviour in the power block

In this section, a comparative study was performed using the referenced and the improved steam behaviour in the power plant block. This comparison addressed the analysis of the principal steam characteristics (steam mass flow and steam pressure) at different points in the power block. The steam behaviour will be explained based on high and low-pressure sections, as demonstrated hereafter.

#### High-pressure turbine section (optimized)

**Figure 17:**
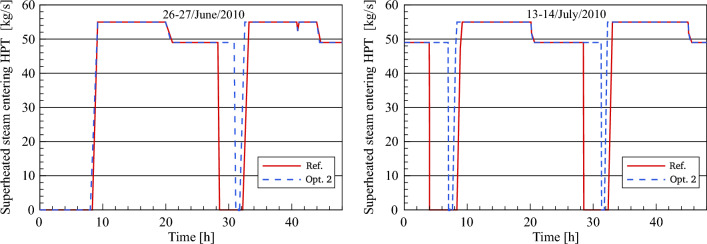
Description of steam mass flow behaviour at HPT inlet.

Figure 17 displays the results obtained by simulation in terms of the dynamic evolution of optimized and referenced superheated steam mass flow through the inlet of the high-pressure turbine for the selected days. The main objective of this comparison is to explore the impact of both methods (single and dual feedwater circuits) on superheated steam production for daytime and evening hours.

In this comparative analysis, a good agreement between the steam mass flow rate in the reference model and optimization 2 can be shown for daytime and night hours. It should be mentioned that the superheated steam in the daytime is produced by the heat exchange between the thermal oil and water/steam through all the heat exchangers in the PB. The power block is operated by means of dual-circuit feedwater in the transient period, where the FWCV_s_ is gradually opening in sync with the gradual closing of three main valves (FWCV_HTF_, the LP PH MCV_HTF_ and the HP PH MCV_HTF_). This, in turn, leads to the production of steam via two lines during the transient period. After sunset, FWCV_HTF_, LP PH MCV_HTF_ and HP PH MCV_HTF_ are fully closed and accordingly the feedwater is heated using the steam extracted from the turbine. As a result, it can be seen here that an improvement in the operational period of the power block is achieved. Thereby the power block provides the superheated steam at a constant mass flow of 55 kg/s for a period ranging between 11.7 and 12.7 h during the daylight and at 49 kg/s for a period of approximately 10–10.5 h during the night. In addition, the superheated steam is produced during the transient period at a mass flow ranging between 49 and 55 kg/s for a period of about 30 min.

**Figure 18:**
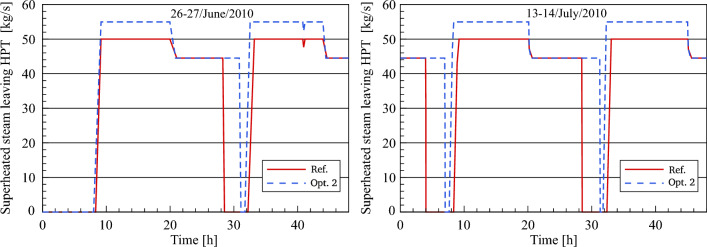
Description of steam mass flow behaviour at HPT outlet.

A comparison is provided of the steam mass flow rate at the HP turbine outlet produced by the optimized model with the reference model's simulated results, plotted in Fig. 18. After comparison, the steam mass flow at the high-pressure turbine outlet in optimization 2 has the same value at the HP turbine inlet (55 kg/s) during daylight. Conversely, the steam mass flow at the HP turbine outlet is equal to 49 kg/s during the night in optimization 2 and in the reference model. This is due to the fact that there is no HTF passed to the second feedwater circuit (feedwater/HTF circuit) and accordingly, the HP preheaters in the first feedwater circuit (feedwater/steam circuit) are operated using the steam instead of the second feedwater circuit. This approach can be used when the demand for energy is low during the night period and the operational period is important more than the amount of electrical power. In contrast to the improved design, the feedwater cycle in the validated plant (FW/S circuit) is operated using steam extractions (5 and 4 kg/s) from the HP turbine and passed the HP-Preheaters (HP-PH_1_ and HP-PH_2_).

**Figure 19:**
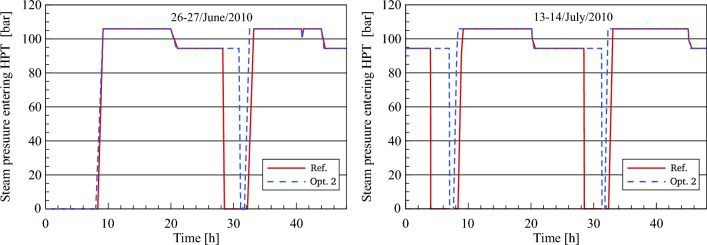
Description of steam pressure behaviour at HPT inlet.

Figure 19 shows a comparison between the optimized and referenced steam pressure. It can be clearly seen how the HPT inlet steam pressure is restricted to the boundary conditions until reaching a design pressure of (106 bar) during the startup and warmup process. As is expected, it can be noticed that the steam pressure during the daytime hours remains unchanged at a value of 106 bars for approximately 40–50 min longer than it is in the reference model. This is because the design inlet HTF temperature (295 °C) in optimization 2 is achieved faster than the design inlet temperature in the reference model. During the evening period, the optimized steam pressure remains constant at a value of 94.42 bars for a period of approximately 10–10.5 h according to the period of steam production. The stability of the optimized steam pressure curves during the night hours for a period longer than the reference model indicated in Fig. 13 reveals the crucial role of the thermal storage energy to accomplish stable steam production.

#### Low-pressure turbine section

**Figure 20:**
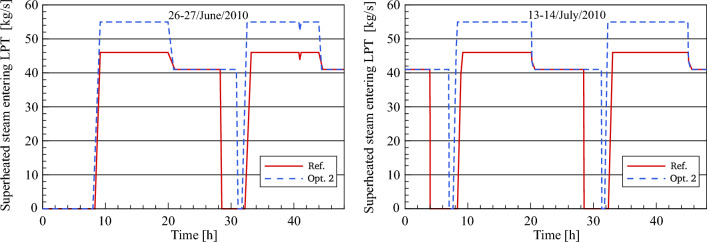
Description of steam mass flow behaviour at LPT inlet.

**Figure 21:**
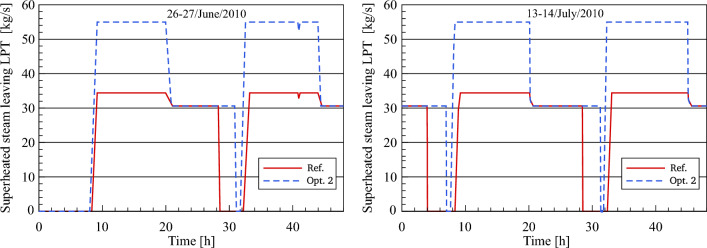
Description of steam mass flow behaviour at LPT outlet.

The improved steam mass flow at the LP turbine inlet and outlet is compared to the numerical results in the reference model, as illustrated in Figs. 20 and 21. The reheated steam mass flow remains unchanged at 55 kg/s throughout the daytime. This is because the feedwater passed through the preheaters is heated using the thermal oil in the feedwater/HTF circuit and accordingly, no steam is taken out of the turbine to the feedwater circuit. During the transient period, the steam mass flow rate in optimization 2 is reduced to the same nominal conditions as the reference model. Obviously, in this period the nominal mass flow of steam in optimization 2 is achieved faster than the referenced mass flow. This is on the one hand due to the greater amount of heat collected in the optimized model and on the other hand, it is applied to the same HTF quantity (600 kg/s). However, there is an additional period of steam production more than the period in the reference model in both periods when the scenario of optimization 2 is used.

**Figure 22:**
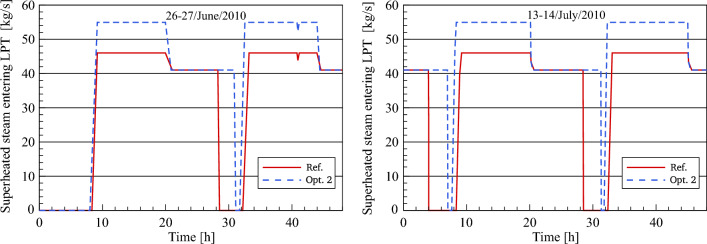
Description of steam pressure behaviour at LPT inlet.

A good agreement between optimized and referenced low-pressure inlet steam pressure can be observed in Fig. 22. Generally, the low-pressure inlet steam pressure behaves the same behaviour as the HP-turbine inlet. In optimization 2, an improvement in the daytime and evening operational periods can be noticed. Further increases in steam temperature and steam pressure are limited by the equipment manufacturer.

## Cost analysis for reference plant and optimized plant

A comparison of the cost of electricity of the referenced PTPP (50MW_el_) and the optimized PTPP (68MW_el_) has been made in this study on the basis of technical specifications and the cost items from the IRENA database, as described in Table 2. Here the optimization process includes increasing the capacity of SF and TSS by about 33% compared to the reference PTPP. The majority of the cost reductions can be obtained for plant balancing, grid attachment, PB, project managing, and improvement costs, as well as these types of expenses, which are practically unchanged for any size of each project^46^. Furthermore, the referenced feedwater circuit (FW/S circuit) was added to the optimised PTPP to obtain longer working hours during the night based on the optimised high storage capacity in order to reduce dependence on fuel during the night period. Generating Levelized Energy Cost (LEC) is an essential metric for measuring the cost of electricity generation. LEC is determined on the basis of the overall cost of a PTPP, distributed by the expected electricity production (kWh) of the system during its service life. The LEC gives an indication of the least cost of electricity expected sold to recover minimally the overall PTPP cost over its useful life. The LEC can be calculated as follows^47^:

1 $$LEC=\frac{crf\times {C}_{invest}+{C}_{annual}}{{E}_{annual}}$$

where $crf$ is the capital recovery factor, ${C}_{invest}$ is the overall plant's investment expenditure, ${C}_{annual}$ is the annual operating and servicing expenses, ${E}_{annual}$ is the annual net electricity production.

**Table 2:** Itemization of capital investment, operating and servicing costs for the net 50MW_el_ and 68MW_el_ PTPPs.

Expenses category	Expenses ($million) to produce 50 MW_el_	Expenses ($million) to produce 68 MW_el_
Overall plant's investment expenditure	364	425.2
Solar field	136.4	180.5
Thermal storage system	38.4	50.6
Power block	20.8	21
Balance of plant ^46^	20.7	20.7
Grid access	10.5	10.5
Site preparation and Infrastructure costs	21.2	25.9
PTPP management	28.1	28.1
PTPP funding	21.8	21.8
PTPP improvement	10.5	10.5
Other expenditures (allowances)	55.6	55.6
O&M expenditures	10.6	12.3

Specifications	Reference PTPP	Optimization 2
Annual electrical production, _net_	180 GWh	245 GWh
Storing duration	7.5 h	10.5 h
Electrical capacitance	50 MW_el_	68 MW_el_
Maximum stored energy	1025 MW_th_ h	1360 MW_th_ h
Levelized energy cost (LEC)	0.248 $/kWh	0.212 $/kWh

A *crf* denotes the relationship of fixed annuity to the present PTPP's entire investment expenses, as computed below:

2 $$crf=\frac{i{\times \left(1+i\right)}^{n}}{{\left(1+i\right)}^{n}-1}$$

where ($i$) actual debt interest level and (n) PTPP lifetime.

The actual debt level and PTPP lifetime for the present research were taken at 8% and 25 years, accordingly.

## Conclusion 

The referenced and optimized (optimization 1) models of the existing PTPP (Andasol II) were developed using APROS software. In the current optimization (optimization 2), improvements have been performed in the optimized feedwater and steam turbine models of the optimized plant model (optimization 1). As demonstrated previously, the PTPP consists of three main parts, namely SF, TSS and PB. As previously explained, the loops of SF are increased from 156 to 208 loops and the capacity of TSS is also raised to 1,360 MW_th_ h to increase the power output and the evening operating time. Here, the referenced feedwater circuit (FW/S circuit) is combined with the feedwater circuit (FW/HTF circuit). The FW/HTF circuit supplies the feedwater during the daytime hours, while FW/HTF circuit is operated during the night. In the sunset period (transient period), the feedwater is supplied to the economizer by both feedwater circuits with different amounts. New regulation circuits are implemented in this optimization 2. Furthermore, the boundary conditions of HTF and steam applied in both circuits are maintained similarly to the referenced and optimized feedwater circuits (optimization 1). The steam turbine is developed by adding regulation valves to the steam extractions for regulating the steam passage during the work of the FW/HTF and FW/S circuits. Hence in the daytime period, the steam turbine is operated with FW/HTF circuit, where the steam flows through the HP-turbine and leaves the LP-turbine with the same quantity because the regulation valves maintain closing until the transient period. Thereafter, the regulation valves are opened during the night period and the steam is extracted into the FW/S circuit to operate as in the reference model. As a result, the electrical power increases when using the same steam turbine and generator of the reference model based on the manufacturer's specifications.

The main conclusions of this optimization are summarised as follows:

- Comparisons were made between the simulated results from optimization 2 for (June 26–27, 2010 and July 13–14 2010) and the reference model, which was validated against data collected at Andasol II. For the optimized model, the outputs exhibit behaviour similar to the results of the validated models, which significantly improves the optimized model findings.
- In the daylight, the steam flows through the HP-turbine and the LP-turbine in the same quantity, while in the evening period, it is different because some steam is extracted to the FW/S circuit.
- In the daytime, the nominal electrical power in optimization 2 is approximately 68 MW_el_ instead of 50 MW_el_ as in the referenced model. In the evening period, the nominal electrical power in optimization 2 is equal to the referenced model (48 MW_el_).
- During daytime hours, the nominal value of electrical power (68MW_el_) can be reached for a period of approximately 40–50 min more than it is in the reference model. It should be noted with interest that the same turbine and generator used in the referenced PTPP can be utilized in optimization 2 for obtaining this improvement in the PTPP performance based on the manufacturer's specifications, as it can produce a power of a maximum of 175 MW.
- In the evening period, the same rate of electrical power production (48 MW_el_) could be accomplished in optimization 2 for a period of approximately 33–40% more than the compensation period in the reference model. As a result, there is less reliance on fossil fuels at night.
- According to the cost analysis, this optimization 2 and the operating strategy followed in this PTPP show that the 16.7% increase in the total costs of the referenced PTPP is justified by a 30% increase in the annual performance. The findings indicate that the specific energy cost of a PTPP is lowered by about 14.5% by increasing the output of the PTPP from 50 to 68 MW_el_.

## Data Availability

The datasets generated during and/or analyzed during the current study are not publicly available as the data also forms part of an ongoing study, but are available from the corresponding author on reasonable request.

## Abbreviations

Apros: Advanced process simulation software
Attemp: Attemperator
AD: Adder
CV: Control valve
DNI: Direct normal irradiation
ECON: Economiser
EVAP: Evaporator
EX CV: Extraction control valve
FW: Feedwater
FWCV_HTF_: Feedwater/HTF control valve
FWCV_S_: Feedwater/steam control valve
HPMSCV: High-pressure main steam control valve
HP-PH: High-pressure preheater
HP PH MCV_HTF_: High-pressure preheater main control valve of HTF
HPT: High-pressure turbine
HTF: Heat transfer fluid
LP: Low pressure
LP Attemp CV: Low-pressure attemperator control valve
LPBPCV: Low-pressure bypass control valve
LPT: Low-pressure turbine
LP-PH: Low-pressure preheater
LP PH MCV_HTF_: Low-pressure preheater main control valve of HTF
$$\dot{m}$$: Mass flowrate
PTPP: Parabolic trough power plant
RDCV: Redirection control valve
RH: Reheater
S: Steam
SF: Solar field
SH: Superheater
T: Temperature
TS: Thermal storage
TS DCV_o_: Thermal storage dual control valve at the outlet
TS MCV_i_: Thermal storage main control valve at the inlet
TSS: Thermal storage system
